# A therapist-administered self-report version of the Walking Index for Spinal Cord Injury II (WISCI): a psychometric study

**DOI:** 10.1038/s41393-024-00985-8

**Published:** 2024-04-02

**Authors:** Marsha Ben, Federica Tamburella, Matteo Lorusso, Joanne V. Glinsky, Keira E. Tranter, Giorgio Scivoletto, Lynn Blecher, Anneliese Harris, Giovanni Galeoto, Joshua Wan, Lisa A. Harvey

**Affiliations:** 1 John Walsh Centre for Rehabilitation Research, University of Sydney, Kolling Institute, Sydney, Australia; 2 Foundation Santa Lucia, Rome, Italy; 3 Link Campus University of Rome, Department of Life Sciences, Health and Health Professions, Rome, Italy; 4 The Prince of Wales Hospital, Sydney, Australia; 5 The Royal North Shore Hospital, Sydney, Australia; 6 Department of Human Neurosciences, Sapienza University of Rome, Rome, Italy; 7 University of Sydney, Sydney, Australia

**Keywords:** Spinal cord diseases, Outcomes research

## Abstract

**Objective:**

To develop a self-report version of the Walking Index for Spinal Cord Injury II (WISCI II) and to test its reliability and validity.

**Study design:**

Psychometric study.

**Setting:**

Spinal cord injury (SCI) rehabilitation centres in Australia and Italy.

**Participants:**

Eighty people with SCI were recruited from a sample of convenience.

**Methods:**

Two self-report versions of the WISCI II were developed. Both versions were administered in English at the Australian site, and in Italian at the Italian site through an online platform. The format of the first self-report version (SR-V1) was similar to the original face-to-face WISCI II. The second self-report version (SR-V2) had more questions, but each question required participants to focus on one aspect of walking at a time. Participants completed SR-V1 and SR-V2 with assistance from research physiotherapists on two separate occasions, three to seven days apart. The original WISCI II was then administered through a face-to-face assessment by an independent physiotherapist. The intra-rater reliability and validity of SR-V1 and SR-V2 were determined with intraclass correlation coefficients (ICC) and percent close agreements.

**Results:**

The data from the Australian and Italian sites were pooled. The validity and reliability of the two self-report versions were very similar, with SR-V2 performing slightly better than SR-V1. The ICC (95% confidence interval) of SR-V2 was 0.87 (0.81–0.92). The ICC reflecting the agreement between the self-report and the face-to-face WISCI was 0.89 (0.84–0.93).

**Conclusion:**

Both versions of the self-report WISCI II provide a reasonable substitute for a face-to-face assessment although therapists preferred SR-V2.

## Introduction

Walking is an important and common goal for people with spinal cord injury (SCI) and a key measure of physical function. Moreover, people with SCI rate walking as a very important determinant of their quality of life [1]. Not surprisingly, most cohort studies and clinical trials interested in some aspect of physical rehabilitation and walking include a measure of walking ability [1].

The most commonly used measure of walking ability is the Walking Index for Spinal Cord Injury Version II (WISCI) [2–6]. WISCI was developed for research purposes [7, 8] and has been validated for use in the acute [9] and chronic [10–12] stages of SCI, with high inter- and intra-rater reliability [7, 9]. It is a hierarchical 21-point scale, where the WISCI score reflects the participants’ ability to walk on a 10-m flat smooth surface. Scores range from 0 (unable to stand and/or participate in assisted walking) to 20 (ambulates with no devices, no braces and no physical assistance, 10 m) [13]. The assessment involves progressing the participants through each level of the scale by manipulating different combinations of walking aids, leg braces and physical assistance, until the maximal score is attained. The WISCI score does not take walking speed and quality of gait into consideration, and the participants are scored on how they can walk in a testing environment, rather than on how they prefer to walk in the community or at home [13].

WISCI assessments can be difficult to conduct for participants living in the community. Either therapists need to travel to the participants, or the participants need to travel back to the clinic. Both options are inconvenient, time-consuming, and costly. Therefore, a self-report version would offer a more practical and cost-effective way of administering WISCI for people living with SCI in the community.

We considered various ways of administering a self-report WISCI. One option was to simply ask the participants how they typically walk in the community, however, this would be potentially problematic, because people with SCI can often walk in several different ways. For example, a person may use a walker and an ankle-foot-orthosis (AFO) in the community (WISCI level 9), walk with a walking stick and an AFO within the home (WISCI level 15), occasionally walk with a walker and no AFO for exercise (WISCI level 13) but may be able to walk unaided and with an AFO for short distances if prompted (WISCI level 18). People with SCI are unlikely to be able to easily identify the combination of aids, orthoses, and assistance that derives the highest WISCI score by merely looking at the scale. So, we designed a self-report Version 1 (SR-V1) which was very similar to the original scale but comprised 21 questions based on each level of the WISCI.

Whilst the SR-V1 is similar to the original WISCI, we were concerned that asking participants to think about all the various combinations of walking that comprise the scale may be confusing. For example, participants may find it confusing to answer repeated and similar questions in which three variables (walking aids, assistance, and braces) are simultaneously manipulated at each level. So, we decided to develop a second version, which we coined self-report Version 2 (SR-V2). This version was more nuanced and only asked relevant and essential questions. The questions comprising the SR-V2 were simpler than the questions in SR-V1 because they only required participants to consider one aspect of walking at a time.

Therefore, the aims of the study were to:

Aim 1: develop two self-report versions of the WISCI, and to test both versions at an Australian and Italian site.

Aim 2: determine and compare the validity and reliability of the two WISCI self-reports with the original face-to-face version.

Aim 3: determine which of the two WISCI self-report versions participants and therapists prefer.

## Methods

### Aim 1: To develop two self-report versions of the WISCI

Two self-report versions of the WISCI scale were initially developed in English. Both versions were then translated to Italian using a cross-cultural adaptation process according to the principles of Good Practice for translation and cultural adaptation [14] (see Supplementary file 1 for details). Participants from the Australian and Italian sites completed the English and Italian versions of the self-report, respectively.

Both SR-V1 and SR-V2 were designed to be administered online through the survey feature of the secure REDCap database with telephone support provided by a research physiotherapist. The overarching goal was to ensure that the WISCI self-reports were user-friendly and easy for people with SCI to complete, whilst capturing maximum scores as close as possible to those attained through a face-to-face assessment. The details of the two self-report versions are described below.

**Fig. 1:**
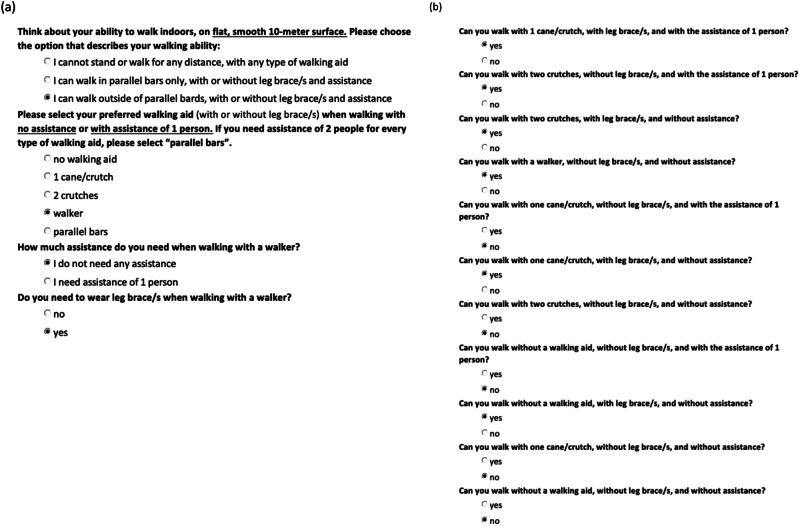
An example of a person’s responses to Version 1 of the self-report WISCI (SR-V1). **a** First part of this self-report involves 4 initial questions that appear one by one to derive the “self-selected” WISCI level. In this example the person has a WISCI level of 9. **b** Once the “self-selected” WISCI is determined, the remaining subsequent yes/no questions appear. The participant answers every remaining question to derive the highest WISCI level. In this example the person has WISCI level 18.

*SR-V1* consists of two parts and involves a series of yes/no questions that correspond to the original WISCI scale (branching logic used to develop SR-V1 is provided in the Supplementary file 2). The first part focuses on how the participants prefer to walk (see Fig. 1a). This level of mobility is often referred to as self-selected WISCI [10]. The second part of SR-V1 involves answering additional yes/no questions, where each question corresponds to the remaining WISCI levels beyond participants’ self-selected level. Each question addresses the three aspects of mobility together (i.e., need for walking aids, orthoses, and assistance). These additional questions prompt the participants to consider all the different ways that they *can* walk and aims to capture their maximal WISCI scores. The participants are required to answer every question and their answers are used to determine their WISCI score. For example, the maximal WISCI self-report score for a person with a self-selected WISCI level 9, and who answers “yes” to questions 13, 15, and 18, and “no” to all other questions, would be 18 (see Fig. 1b).

*SR-V2* also poses a series of yes/no questions considering one aspect of walking ability at a time (see www.wisci.org). A branching logic algorithm was developed where a yes/no question about the use of a specific walking aid is followed by a yes/no question about the need for assistance, and then a question about the need for leg braces, and so on. The sequence of questions within the branching logic algorithm is based on the original WISCI scale (branching logic used to develop SR-V2 is provided in the Supplementary file 3).

**Fig. 2:**
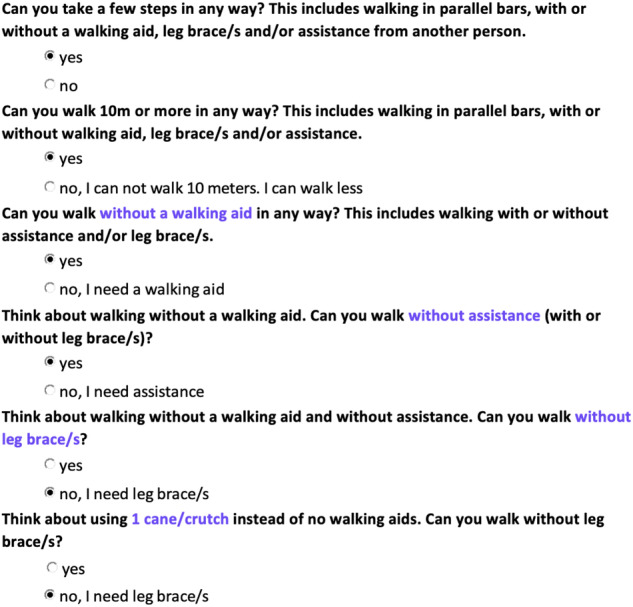
Responses to Version 2 of the self-report WISCI (*SR-V2*) using the same example as the one used to depict Version 1 (SR-V1) (Fig. 1). Questions are revealed one at a time, based on the answer, using a branching logic. Each question addresses one aspect of walking at a time. Additional yes/no questions continue to appear until the highest level of WISCI is reached (score = 18).

The initial two questions address the walking ability of people who are unable to stand/step, and those that can walk for less than 10 m in parallel bars. From there, the questions “branch” into five main question sequences, each branch relating to walking along a 10-m distance with a different type of walking aid (namely no walking aid, 1 cane/crutch, 2 crutches, walker, and parallel bars). Depending on their answers, the participants continue to be systematically presented with additional questions that address their use of different types of walking aids, level of assistance, and need for leg braces, until a maximal WISCI level is reached within the branching logic. For the example used above, where the person’s mobility ranges between the minimal WISCI level 9 and the maximal level 18, the sequence of SR-V2 questions is demonstrated in Fig. 2.

When developing SR-V2, every possible combination of walking aids/level of assistance/need for leg braces was catered for in the various series of different questions. The challenge of developing this self-report version was to ensure that every participant attained a maximal score. Ultimately, our branching logic algorithm required 322 different combinations of 64 unique questions to ensure that any person could attain a maximal score irrespective of whether he or she can walk in one or in several different ways. Each combination contained between 1 and 17 questions in a unique series with a median (IQR) of 8 (6 to 9) questions required to derive a maximal score.

To ensure that the different combinations of questions were exhaustive and covered every possible combination of walking ability, we created a simulation model using statistical software (STATA V16). The simulation model enabled us to develop 7740 unique pseudo people all walking in up to 5 different ways after manipulating types of aids (no walking aid, 1 cane/crutch, 2 crutches, walker, and parallel bars), levels of assistance (no assistance, assistance of 1 person, assistance of 2 people), and use of orthoses (with and without). The real maximal WISCI score was then derived for each pseudo person. Then each pseudo person was put through our branching algorithm (which only allowed yes or no answers to our different combinations of questions). This simulation model confirmed that the algorithm derived the appropriate maximal scores for each person.

### Aim 2: To determine and compare the validity and reliability of the two self-report versions of the WISCI

Eighty individuals with SCI were recruited from a sample of convenience from the Australian and Italian sites. Ethical approvals were provided by the relevant committees, and written informed consent was obtained from all participants. We reasoned that 80 participants would provide good precision around the estimates of reliability and validity. Participants over 18 years of age, and those speaking sufficient English (in Australia) and Italian (in Italy) were included regardless of their neurological level of injury (as per the International Standards for Neurological Classification of SCI: ISNCSCI) or time since injury. Participants with recent injuries (less than 6 months duration) were recruited once their treating therapists confirmed that their mobility status was stable and had not changed for at least one week. Those with cognitive or medical conditions precluding them from understanding or cooperating were excluded from the study. Recruitment of participants with no ability to walk (i.e., WISCI level 0) or full ability to walk (i.e., WISCI level 20) was limited to four per recruitment site. This was done to minimise clumping at each extreme of the scale and to ensure that reliability and validity of the full spectrum of the WISCI was explored.

The participants were asked to complete the two WISCI self-report versions online on two separate occasions, three to seven days apart. The order in which the participants completed SR-V1 and SR-V2 on day 1 was randomised, however, it was then kept the same on day 2. A research physiotherapist, unaware of participants’ walking ability, provided telephone (and occasionally in-person) support on both days to assist people with poor hand function or those who had difficulties using the REDCap software. Furthermore, support was provided to ensure that the participants understood the questions and to clarify any terminology or wording (e.g., leg braces, use of walking aid, and assistance). The researcher had a copy of the participants’ most recent LEMS and used clinical reasoning to query participants during administration of the self-report if participants’ answers seemed to contradict their motor scores. Within a week of completing their self-reports, the participants had a face-to-face WISCI assessment. The time between the two assessments was kept to a minimum to reduce the likelihood of any real change in participants’ walking abilities. The face-to-face assessments were administered in the clinical setting by trained physiotherapists. The physiotherapists administered the face-to-face WISCI according to the WISCI guide, ensuring that every participant’s mobility was progressed through the scale until a maximal level was attained [13]. Neither the physiotherapist nor the participants were aware of the participants’ self-reported scores.

Data were collected and stored using a secure REDCap database and analysed using STATA V16. Intra-rater reliability for SR-V1 and SR-V2 was determined by comparing the results attained on the first testing day with those attained on the second testing day. Validity for each of the two self-report versions was calculated by comparing the mean results from day 1 and day 2 with the results attained from the face-to-face WISCI assessments. ICC values of > 0.75 were interpreted as reflective of excellent reliability, 0.4–0.75 as fair to good, and < 0.4 as poor [15]. The intra-rater reliability and validity of the two versions of the self-report WISCI were determined using Intraclass Correlation Coefficient (ICC_3,1_) and Bland-Atman plots. In addition percent close agreements [16–18] were calculated to determine the number of times (expressed as a percentage) two scores were identical, or within 1, 2, or 3 points of each other.

### Aim 3: To determine which self-report WISCI version the participants and therapists preferred

Participants’ preferences for SR-V1 and SR-V2 were captured immediately after each assessment by asking them to rate “*how difficult*” and “*how time-consuming*” they found each version on a 11-point Global rating scale [19]. The two scales were anchored at each end (from 0 to 10) with the phrases “*very easy*”/“*very quick*”, and “*very difficult*”/“*very time-consuming*”, respectively.

The research therapists were also asked about which self-report version they preferred and would use in the future if given the option. They were asked to consider the ease of administration, the time required and the amount of prompting that participants needed to understand the questions.

## Results

The demographic characteristics and neurological features of the participants (*N* = 80) are shown in Table 1. All participants completed the self-report and the face-to-face WISCI assessments. Thirty-nine participants were recruited from the Australian site and 41 participants were recruited from the Italian site. Participants from the Australian and Italian sites completed the English and Italian versions of the self-report, respectively. The data from the English and Italian sites were pooled (*n* = 80) because (i) there was no reason to believe that the translation of simple sentences about walking aids, orthoses or assistance could be interpreted differently in Italian than in English (particularly when the translated version was back translated); (ii) a post-hoc analysis by language (performed on the request of a reviewer) did not indicate any notable differences between the 2 languages with a slight suggestion that the Italian version was more reliable than the English version allaying any concerns that the translated version was not a good reflection of the original English version, and (iii) pooling the data ensured that the point estimates reflecting reliability and validity would have good precision (please see Supplemental file 4 for separate data analysis).

**Table 1:** Participant characteristics (*N* = 80).

Age (years), median (IQR)	59 (43 to 68)
Time since injury (months), median (IQR)	9.1 (3.6 to 67.4)
Sex, *n* (%)	
Male	55 (69%)
Female	25 (31%)
Type of spinal cord injury, *n* (%)	
Tetraplegia	37 (46%)
Paraplegia	43 (54%)
ASIA Impairment Scale classification, *n* (%)	
A	5 (6%)
B	1 (1%)
C	18 (23%)
D	56 (70%)
Lower Extremity Motor Score, median (IQR)	35 (28 to 41)
Receiving physiotherapy, *n* (%)	
Inpatient	45 (56%)
Outpatient/community-based	20 (25%)
No therapy	15 (19%)
WISCI scores, *n* (%)	
0–5	12 (15%)
6–10	12 (15%)
11–15	11 (14%)
16–20	45 (56%)
WISCI scores, median (IQR)	17 (8 to 19)

Legend: *WISCI* Walking Index for Spinal Cord Injury, *ASIA* American Spinal Injury Association. The WISCI scores were attained from the face-to-face assessment using the original WISCI.

**Fig. 3:**
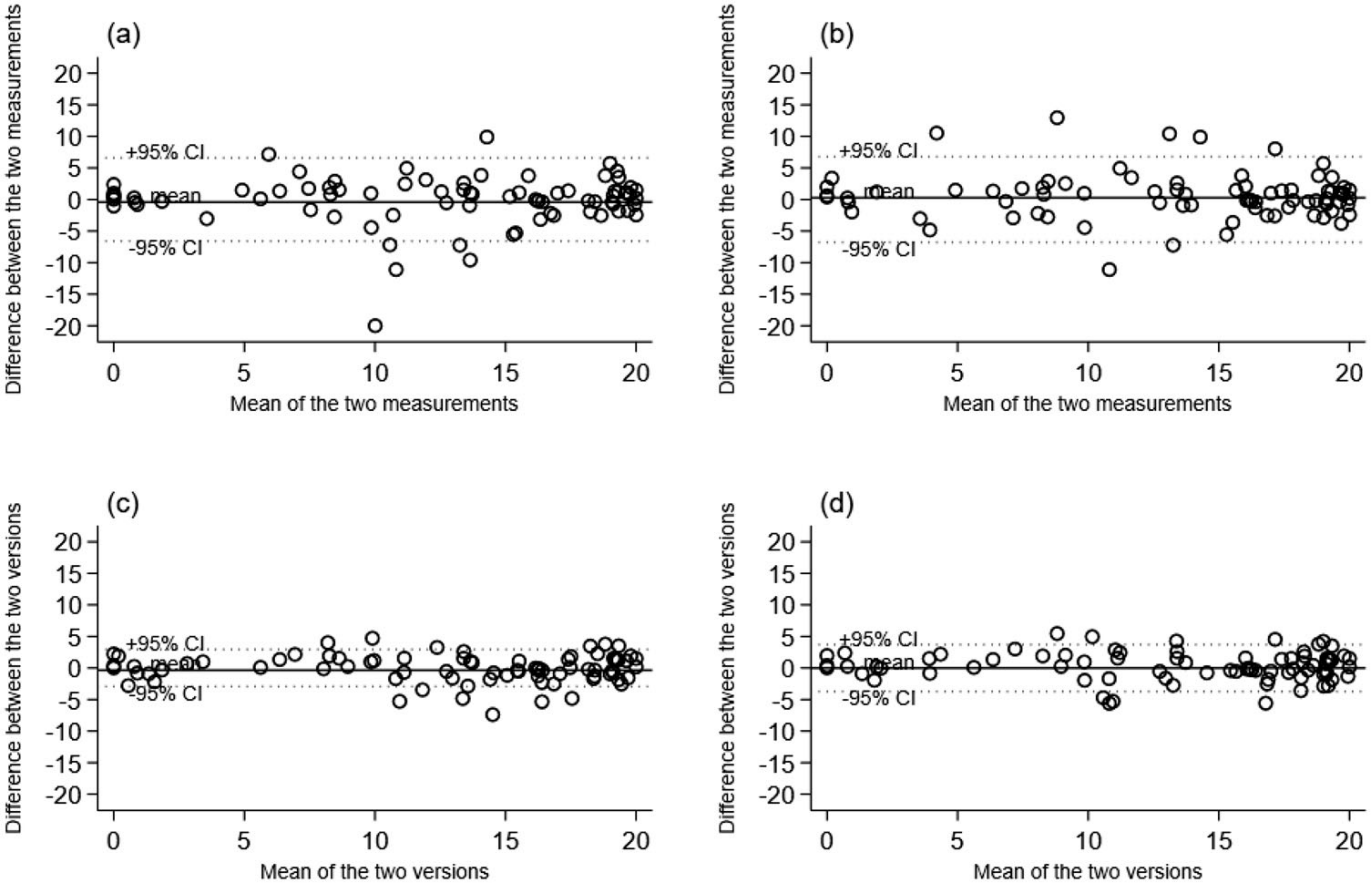
Bland-Altman plots show the levels of agreement between SR-V1 and SR-V2 scores obtained on two separate testing days, as well as the levels of agreement between self-report and face-to-face version of the WISCI. Bland-Altman plots show the level of agreement between **a** SR-V1 measurements obtained on day 1 vs. day 2; **b** SR-V2 measurements obtained on day 1 vs. day 2; **c** SR-V1 (mean of the results obtained on day 1 and day 2) and face-to-face WISCI versions; **d** SR-V2 (mean of the results obtained on day 1 and day 2) and face-to-face WISCI versions.

Intraclass Correlation Coefficients (95% confidence interval; CI) for the repeat assessments of SR-V1 and SR-V2 were 0.86 (0.79 to 0.91) and 0.87 (0.81 to 0.92), respectively, demonstrating excellent intra-rater reliability of both versions. The percent close agreements are shown in Table 2 [16]. The scores obtained on day 1 and day 2 were within three points of each other 82% of time for SR-V1, and 84% of the time for SR-V2. The level of agreement between self-reports obtained on testing day 1 and day 2 for SR-V1 and SR-V2, reflecting intra-rater reliability of each version, are also depicted in the Bland-Altman plots (Fig. 3a and b). For both self-report versions, mean differences between day 1 and day 2 are almost equal to zero and most values are represented within the limits of agreement. (Fig. 3a and b).

**Table 2:** Percent close agreement between self-report measurements collected on day 1 and day 2.

Difference (points)	SR-V1	SR-V2
0	58%	58%
1	71%	67%
2	76%	70%
3	82%	84%
4	87%	90%
5	90%	94%

The Intraclass Correlation Coefficient (95% CI) comparing the self-reports with the face-to-face WISCI was 0.90 (0.85 to 0.93) for SR-V1, and 0.89 (0.84 to 0.93) for SR-V2, demonstrating excellent validity of both versions of the self-reports compared to the original WISCI scale. The percent close agreement is shown in Table 3. Face-to-face WISCI scores were within 3 points of the scores obtained with SR-V1 78% of the time, while they were within 3 points of SR-V2 scores 82% of the time. The level of agreement between the face-to-face WISCI and SR-V1 or SR-V2 are shown in the Bland-Altman plot (Fig. 3c and d). The mean differences between SR-V1 and face-to-face WISCI is zero, suggesting that the results obtained from the two measurements were almost coincident. For the comparison between SR-V2 and the face-to-face WISCI, the mean difference is slightly less than zero indicating that the results obtained with SR-V2 are slightly lower than with the original version. There are no substantial differences between the number of outliers (i.e., cases of disagreement) for SR-V1 and SR-V2.

**Table 3:** Percent close agreement between the self-report measurement for SR-V1 and SR-V2 (mean of scores obtained on day 1 and day 2) and face-to-face WISCI scores.

Difference (points)	SR-V1 vs. Face-to-face	SR-V2 vs. Face-to-face
0	39%	44%
1	57%	54%
2	68%	72%
3	78%	82%
4	82%	84%
5	92%	91%

The participants found both versions very easy and very quick to complete as indicated by the 11-point rating scales. The median (IQR) score for the ease of use was 1.0 (IQR 0.0 to 3.0) for both SR-V1 and SR-V2, where a score of 0 indicates “very easy”. The median score for the time taken to complete each self-report was 1.0 (IQR 0.0 to 3.0), where 0 indicates “very quick”. The SR-V2 was slightly shorter based on the number of questions that the participants needed to answer to determine their maximum WISCI scores, answering a median (IQR) of 8 (6 to 9) questions instead of 11 (5 to 14) questions required to complete SR-V1. It took participants a median of 2 min (IQR 2 to 3) to complete SR-V1 and 3 min (IQR 2 to 4) to complete SR-V2.

The therapists preferred SR-V2 and indicated that they would use this version in the future if given the option. They found it easier and quicker to administer and did not require as many prompts from them to help participants understand the questions as SR-V1.

## Discussion

In this psychometric study, two self-report versions of the WISCI were developed and tested for reliability and validity. Based on the ICC values, both self-report versions have excellent intra-rater reliability and validity (ICC > 0.75) [15]. The participants rated both self-reports as equally easy and quick to use but the therapists preferred SR-V2. Furthermore, SR-V2 required fewer questions to be answered to attain a maximal WISCI self-report score.

Whilst the ICC values indicate excellent reliability of the two self-report versions of the WISCI, the results of the percent close agreement are less favourable (see Table 2). For example, only 84% of the repeat SR-V2 measurements were within 3 points of each other. This contrasts the results of two studies on the face-to-face WISCI that found that repeat results were within 3 points of each other at least 92% of time [9, 11]. This suggests that the original face-to-face WISCI scale has better reliability than the two self-report versions. However, these findings and comparisons need to be interpreted with caution. The two studies on the face-to-face WISCI had small samples (26 participants in one study [11], and 33 in the other [9]). More importantly, 30% of participants in these 2 studies mobilised at a WISCI level 20, potentially leading to clustering of data at the end of the scale where the agreement between assessments is likely to be higher. We capped the number of people at the top and the bottom of the scale to avoid this potential problem and to ensure we did not artificially inflate our measures of reliability and validity. We reasoned that it would be far easier for people to accurately and reliably self-report no or full ability to walk than to self-report their abilities to walk with different combinations of aids, orthoses, and assistance. This proved to be true as evident by the less variability in the spread of data at the two extremes of the scale on the Bland–Altman plots, and with more spread in the middle of the scale (see Fig. 3). The lower level of agreement in the middle of the scale is in part a function of ordinal scales where variations at the top and bottom of the scale can only be one directional but is also a function of the WISCI scale. That is, regardless of whether the WISCI is administered face-to-face or not, the definitions of no ability to walk and full ability to walk are not ambiguous. So, the inclusion of more participants at the top of the scale in the two studies looking at the reliability of the face-to-face WISCI compared to our study could alone explain the differences. A future study needs to look at the reliability of the face-to-face and the self-report version in the same group of participants to ensure that any comparison is valid.

One of the most likely sources of error with the SR-V1 or SR-V2 is participants’ interpretation of levels of assistance required to safely walk 10 m. Participants need to differentiate between needing no assistance and needing assistance of one person to walk, with the distinction between the two making a notable difference to the WISCI scores. For example, the difference between walking with a walker with and without assistance is the difference between a score of 8 and 13. The equivalent differences when walking with two crutches is 11 and 16; and walking with one cane/crutch is 14 and 19. Yet decisions about the amount of assistance required is highly subjective and participants are likely to be influenced by their confidence, previous falls, amount of practice with a therapist and their time since injury. It is particularly difficult for participants to know whether they require contact guarding (which is defined as assistance) [13]. Even therapists differ in their judgement about when contact guarding is or is not required. Regardless, researchers need to ensure that participants understand the definitions and appreciate that contact guarding is a form of assistance. This may require ongoing prompting from researchers during the administration of the self-report WISCI (as provided in this study).

There may be differences in the reliability and validity of the self-report WISCI in different types of people. If we could identify those that are better at self-reporting, then the self-report version could be used for these people and the face-to-face version for others. The most obvious variable that may influence reliability is time since injury. So, those who have had their injuries for longer periods of time may be better at self-reporting than those who are only recently injured. Presumably, those with injuries for many years have a clear understanding of how they can and cannot walk. Conversely, people with recent injuries have other advantages that make it easier for them to self-report than those with established injuries. For example, they may have more opportunities to try different combinations of aids, orthoses, and assistance to appreciate what they may be able to do under a testing situation in the clinic. In addition, those in a hospital setting may have more opportunities to attempt to stand and walk in parallel bars. This may not be the case for a person in the community. A participant’s ability to distinguish between how they can walk in the clinic from how they routinely walk in the community is important because the WISCI score should reflect the former rather than the latter. Post-hoc sub-group analysis to explore whether time since injury was a factor showed that people who sustained their injuries more than 6 months previously (*n* = 36) and those with injuries within 6 months (*n* = 44) had similar levels of agreement between the SR-V2 and the face-to-face WISCI. However, there was a slight tendency for people with recent injuries to have a better correlation and agreement of the SR-V1 with the face-to-face WISCI (ICC (95% CI) = 0.93 (0.87 to 0.96)), than for those with long-standing injuries (ICC (95% CI) = 0.85 (0.73 to 0.92)). These results need to be interpreted with caution as there are other factors that may explain this slight difference.

It is possible that some of the differences between the WISCI measures taken on different days reflected real changes in participants’ abilities to walk. We tried to minimise this possibility by reducing the time between repeat assessments. For example, the two self-report WISCI assessments were completed within a median of 5 days (IQR 3-8 days) of each other, and the face-to-face assessments were completed within 7 days (IQR 7–9 days) of the first self-report. We also tried to reduce the risk of real change in participants’ abilities to walk by only enroling participants whose walking abilities had largely stablised. For example, those with recently-acquired SCI were only included if their treating physiotherapists confirmed that their mobility status had not changed for at least a week. Nonetheless, it is possible that some of the differences between repeat measures reflect a real change in participants’ ability to walk thereby underestimating our measures of reliability and validity. This was more likely to be the case in those with recent injuries (i.e., those who sustained their injuries less than 6 months prior) than those who sustained their injuries more than 6 months prior. Yet, our post-hoc analysis (comparing results <6 months with >6 months) do not suggest this is the case.

We found that many people required assistance either to use the online system or to understand the questions. For example, several people who could walk with a walker answered “*no*” to the question: “*Can you walk 10* *m or more in any way*?”. When prompted by the therapist, it became clear that they did not perceive walking with a frame as walking per se. And some people were not familiar with terms such as walking aids, walkers, and leg braces. These types of queries needed to be explained by the therapist over the telephone, requiring more explanation than could be put in the written instructions. Perhaps some of these issues could be addressed with further refinement of the two versions of the self-report WISCI. But regardless people are probably going to have problems using any online system or the App on their own because the scale is inherently repetitious and dependent on the manipulation of three variables in different ways (aids, orthoses, and assistance). We found that it required the researchers to encourage people to give each question considerable attention to fully understand what was being asked.

In conclusion SR-V1 and SR-V2 have acceptable intra-rater reliability and validity. Both versions will introduce slight error compared to the original face-to-face assessment. These can probably be minimised with careful support directed at ensuring participants understand the definitions of walking and assistance. Further refinement of the questions may also help although care needs to be taken to ensure the instructions are not overly detailed and technical. Regardless, researchers need to weigh up the small sacrifice in accuracy with the self-report version of the WISCI with the sometimes insurmountable problems and cost of conducting face-to-face WISCI assessments. Our results indicate that in most situations either version of the self-report WISCI will provide a reasonable substitute for a face-to-face assessment but that therapists slightly preferred SR-V2.

### Supplementary information

Supplementary file 1

Supplementary file 2

Supplementary file 3

Supplementary file 4

## Data Availability

The data generated and/or analysed during this study are available from the corresponding author on reasonable request.
