# Supplementary material for: A therapist-administered self-report version of the Walking Index for Spinal Cord Injury II (WISCI): a psychometric study
Source: Spinal Cord. 2024 Apr 2;62(6):307–13. doi: 10.1038/s41393-024-00985-8 (PMC11199132; doi:10.1038/s41393-024-00985-8)
Supplement: Supplementary file 1 [file 41393_2024_985_MOESM1_ESM.pdf]

The Translation and Cross-Cultural Adaptation Process used to adapt WISCI self-reports from English to Italian Language.

This is a supplement to the main paper titled *A self-report version of the Walking Index for Spinal Cord Injury II (WISCI): a psychometric study.* The supplemental file provides a summary of the Translation and Cross-Cultural Adaptation process that was adhered to for the translation of the two self-report WISCI versions from English to Italian languages. The process was developed according to Principles of Good Practice [Wild, Grove et al, 2005]. The team members involved in the various steps of the translation and adaptation process were Tamburella F (key in country person), Lorusso M (forward translator), and Scivoletto G (project manager and a back translator native speaker) from Foundation Santa Lucia of Rome.

The table below summarises the critical components of each step involved in the process:

**Steps taken**	**Critical components of each step**
Preparation	Permission was obtained from the developer of the to use and translate the WISCI self-report V1 and V2 from English to Italian language. It was ensured that the conceptual basis of the WISCI self-report V1 and V2 were captured in the translation.
Forward Translation	Two independent translations from English (original source) to Italian (target) language were developed for WISCI self-report V1 and V2.
Reconciliation	The two independent translations were compared and merged into a single forward translation of WISCI self-report V1 and V2.
Back translation	WISCI self-report V1 and V2 were translated from the target Italian Language version back to English. This was done to provide quality-control and to demonstrate that the meaning of the Italian translation remained correct once WISCI self-reports were translated back to source English language.
Back translation review	Comparison of the back-translated versions with the original to highlight and address discrepancies, which is then revised in the process of resolving the issues.
Harmonization	The independent translations of each WISCI self-report were compared with each other. Discrepancies between the back-translations and the original WISCI self-report in English were reviewed and rectified.
Cognitive debriefing	The newly translated WISCI V1 and V2 self-reports were tested on a small sample of 5 Italian patients with SCI. Alternative wording, understandability, interpretation, and cultural relevance of the translation were tested during this stage of the process.

Review of cognitive debriefing results and finalization	Final changes were made to the Italian translation of WISCI self-reports V1 and V2 following the cognitive debriefing stage of the process.
Proof reading and final report	Final corrections were made during the proofreading stage of the process and a report was produced describing the Translation and adaptation of the WISCI self-report V1 and V2.

The final adapted self-report WISCI versions were used at the Italian site.

References:

1. Wild D, Grove A, Martin M, Eremenco S, McElroy S, Verjee-Lorenz A *et al.* Principles of Good Practice for the Translation and Cultural Adaptation Process for Patient-Reported Outcomes (PRO) Measures: Report of the ISPOR Task Force for Translation and Cultural Adaptation. *Value in health* 2005; **8**(2)**:** 94-104.
