# Supplementary material for: A therapist-administered self-report version of the Walking Index for Spinal Cord Injury II (WISCI): a psychometric study
Source: Spinal Cord. 2024 Apr 2;62(6):307–13. doi: 10.1038/s41393-024-00985-8 (PMC11199132; doi:10.1038/s41393-024-00985-8)
Supplement: Supplementary file 2 [file 41393_2024_985_MOESM2_ESM.pdf]

Supplementary file 2: Self-report Version 1

Table 1: Part 1 of SR-V1: *self-selected* WISCI. The questions and answers with corresponding coding are presented. A branching logic indicates when a question is displayed. For example, if a person chooses option #1 to question 1 (i.e., "I can walk in parallel bars only…"), then question 2 is displayed. If a person chooses option #2 to question 1 (i.e., "I can walk outside of parallel bars…"), then question 3 is displayed. The scoring is determined by the scoring matrix (see Table 2).

No	Question	Answers	Branching logic
q1	Think about your ability to walk indoors, on flat, smooth 10-meter surface. Please choose the option that describes your walking ability:	**0:** cannot stand or walk for any distance, with any type of walking aid<br>**1:** I can walk in parallel bars only, with or without leg brace/s and assistance<br>**2:** I can walk outside of parallel bars, with or without leg brace/s and assistance	
q2	Can you walk at least 10 meters in parallel bars?	**0:** yes, I can walk 10 meters<br>**1:** no, I cannot walk 10 meters. I can take a few steps.	[q1] = '1'
q3	Please select your preferred walking aid (with or without leg brace/s) when walking with no assistance or with assistance of 1 person. If you need assistance of 2 people for every type of walking aid, please select "parallel bars".	**0:** no walking aid<br>**1:** 1 cane/crutch<br>**2:** 2 crutches<br>**3:** walker<br>**4:** parallel bars	[q1] = '2'
q4	How much assistance do you need when walking between parallel bars (with or without leg brace/s)?	**0:** I do not need any assistance<br>**1:** I need assistance of 1 person<br>**2:** I need assistance of 2 people	[q2] = '0' or [q3] = '4'
q4a	Do you need to wear leg brace(s) when walking in parallel bars?	**1:** yes<br>**0:** no	[q4] = '0' or [q4] = '1' or [q4] = '2'
q5	How much assistance do you need when walking with a walker?	**0:** I do not need any assistance<br>**1:** I need assistance of 1 person	[q3] = '3'
q5a	Do you need to wear leg brace/s when walking with a walker?	**1:** yes<br>**0:** no	[q5] = '0' or [q5] = '1'
q6	How much assistance do you need when walking with 2 crutches?	**0:** I do not need any assistance<br>**1:** I need assistance of 1 person	[q3] = '2'
q6a	Do you need to wear leg brace/s when walking with 2 crutches?	**1:** yes<br>**0:** no	[q6] = '0' or [q6] = '1'
q7	How much assistance do you need when walking with 1 cane/crutch?	**0:** I do not need any assistance<br>**1:** I need assistance of 1 person	[q3] = '1'

q7a	Do you need to wear leg brace/s when walking with 1cane/crutch?	**1:** yes<br>**0:** no	[q7] = ‘0’ or [q7] = ‘1’
q8	How much assistance do you need when walking without a walking aid?	**0:** I do not need any assistance<br>**1:** I need assistance of 1 person	[q3] = ‘0’
q8a	Do you need to wear leg brace/s when walking without a walking aid?	**1:** yes<br>**0:** no	[q8] = ‘0’ or [q8] = ‘1’

Table 2: The scoring matrix for SR-V2 (see Table 1 for the questions)

Score (self-selected)	Criteria
0	[q1] ='0'
1	[q2] ='1'
2	[q4] ='2' and [q4a] ='1'
2	[q4] ='2' and [q4a] ='0'
3	[q4] ='1' and [q4a] ='1'
4	[q4] ='1' and [q4a] ='0'
5	[q4] ='0' and [q4a] ='1'
5	[q4] ='0' and [q4a] ='0'
6	[q5] ='1' and [q5a] ='1'
7	[q6] ='1' and [q6a] ='1'
8	[q5] ='1' and [q5a] ='0'
9	[q5] ='0' and [q5a] ='1'
10	[q7] ='1' and [q7a] ='1'
11	[q6] ='1' and [q6a] ='0'
12	[q6] ='0' and [q6a] ='1'
13	[q5] ='0' and [q5a] ='0'
14	[q7] ='1' and [q7a] ='0'
15	[q7] ='0' and [q7a] ='1'
16	[q6] ='0' and [q6a] ='0'
17	[q8] ='1' and [q8a] ='0'
17	[q8] ='1' and [q8a] ='1'
18	[q8] ='0' and [q8a] ='1'
19	[q7] ='0' and [q7a] ='0'
20	[q8] ='0' and [q8a] ='0'

Table 3: Part 2 of SR-V1: *maximal* WISCI. All the remaining levels of the WISCI that are higher than the self-selected level (i.e., level identified in part 1) are displayed as yes/no questions. Every question has to be answered. The highest level with a "yes" answer corresponds to the *maximal WISCI* level.

Score (maximal)	Question	Answer
2	Can you walk 10 meters in parallel bars, with leg brace/s, and with the assistance of 2 people?	• yes<br>• no
3	Can you walk in parallel bars with leg brace/s and with the assistance of 1 person?	• yes<br>• no
4	Can you walk in parallel bars, without leg brace/s, and with assistance of 1 person?	• yes<br>• no
5	Can you walk in parallel bars, with leg brace/s, and without assistance?	• yes<br>• no
6	Can you walk with a walker, with leg brace/s, and with the assistance of 1 person?	• yes<br>• no
7	Can you walk with 2 crutches, with leg brace/s, and with the assistance of 1 person?	• yes<br>• no
8	Can you walk with a walker, without leg brace/s, and with the assistance of 1 person?	• yes<br>• no
9	Can you walk with a walker, with leg brace/s, and without assistance?	• yes<br>• no
10	Can you walk with 1 cane/crutch, with leg brace/s, and with the assistance of 1 person?	• yes<br>• no
11	Can you walk with two crutches, without leg brace/s, and with the assistance of 1 person?	• yes<br>• no
12	Can you walk with two crutches, with leg brace/s, and without assistance?	• yes<br>• no
13	Can you walk with a walker, without leg brace/s, and without assistance?	• yes<br>• no
14	Can you walk with one cane/crutch, without leg brace/s, and with the assistance of 1 person?	• yes<br>• no
15	Can you walk with one cane/crutch, with leg brace/s, and without assistance?	• yes<br>• no
16	Can you walk with two crutches, without leg brace/s, and without assistance?	• yes<br>• no
17	Can you walk without a walking aid, without leg brace/s, and with the assistance of 1 person?	• yes<br>• no
18	Can you walk without a walking aid, with leg brace/s, and without assistance?	• yes<br>• no
19	Can you walk with one cane/crutch, without leg brace/s, and without assistance?	• yes<br>• no
20	Can you walk without a walking aid, without leg brace/s, and without assistance?	• yes<br>• no
