# Supplementary material for: A therapist-administered self-report version of the Walking Index for Spinal Cord Injury II (WISCI): a psychometric study
Source: Spinal Cord. 2024 Apr 2;62(6):307–13. doi: 10.1038/s41393-024-00985-8 (PMC11199132; doi:10.1038/s41393-024-00985-8)
Supplement: Supplementary file 3 [file 41393_2024_985_MOESM3_ESM.pdf]

Supplementary file 3: Self-report Version 2

Table 1: The questions and answers for SR-V2 where "no" is coded as "0", and "yes" is coded as "1". The branching logic indicates when a question is displayed. For example, if a person answer "yes" to question 1, then question 2 will be displayed. The scoring is determined by the scoring matrix (see Table 2).

No.	Question	Answers	Branching Logic
q1	Can you take a few steps in any way? This includes walking in parallel bars, with or without a walking aid, leg brace/s and/or assistance from another person.	• yes<br>• no	
q2	Can you walk 10m or more in any way? This includes walking in parallel bars, with or without walking aid, leg brace/s and/or assistance.	• yes<br>• no, I cannot walk 10 meters. I can walk less.	[q1] ='1'
**For all remaining questions, think about your ability to walk safely indoors on a flat, smooth, 10-meter surface.**			[q2] ='1'
q3	Can you walk without a walking aid in any way? This includes walking with or without assistance and/or leg brace/s.	• yes<br>• no, I need a walking aid	
q4	Can you walk with 1 cane/crutch in any way? This includes walking with or without assistance and/or leg brace/s.	• yes<br>• no	[q3] ='0'
q5	Can you walk with 2 crutches in any way? This includes walking with or without assistance and/or leg brace/s.	• yes<br>• no	[q4] ='0'
q6	Can you walk with a walker in any way? This includes walking with or without assistance and/or leg brace/s.	• yes<br>• no	[q5] ='0'
q7	Can you walk in parallel bars in any way? This includes walking with or without assistance and/or leg brace/s.	• yes<br>• no	[q6] ='0'
q8	Can you take a few steps in parallel bars in any way? This includes walking with or without assistance and/or leg brace/s.	• yes<br>• no	[q2] ='0'
q9	Think about walking without a walking aid. Can you walk without assistance (with or without leg brace/s) ?	• yes<br>• no, I need assistance	[q3] ='1'
q10	Think about walking without a walking aid. Can you walk with assistance of 1 person (with or without leg brace/s)?	• yes<br>• no, I need assistance of 2 people	[q9] ='0'
q11	Can you walk with 1 cane/crutch in any way, instead of walking with no aids ? This includes walking with or without assistance and/or leg brace/s.	• yes<br>• no	[q10] ='0'
q12	Can you walk with 2 crutches in any way? This includes walking with or without assistance and/or leg brace/s.	• yes<br>• no	[q11] ='0'
q13	Can you walk with a walker in any way? This includes walking with or without assistance and/or leg brace/s.	• yes<br>• no	[q12] ='0'
q14	Can you walk in parallel bars in any way? This includes walking with or without assistance and/or leg brace/s.	• yes<br>• no	[q13] ='0'
q15	Think about walking without a walking aid, and with assistance of 1 person. Can you walk without leg brace/s?	• yes<br>• no, I need brace/s	[q10] ='1'

No.	Question	Answers	Branching Logic
q11a	Can you walk with 1 cane/crutch instead of no walking aids? This includes walking with or without assistance and/or leg brace/s.	• yes<br>• no	[q15] ='0'
q12a	Can you walk with 2 crutches in any way? This includes walking with or without assistance and/or leg brace/s.	• yes<br>• no	[q11a] ='0'
q13a	Can you walk with a walker in any way? This includes walking with or without assistance and/or leg brace/s.	• yes<br>• no	[q12a] ='0'
q14a	Can you walk in parallel bars in any way? This includes walking with or without assistance and/or leg brace/s.	• yes<br>• no	[q13a] ='0'
q19	Think about using 1 cane/crutch instead of no walking aid. Can you walk without assistance (with or without leg brace/s) ?	• yes<br>• no, I still need assistance	[q15] ='1'
q20	Think about walking with 1 cane/crutch and without assistance. Can you walk without leg brace/s?	• yes<br>• no, I need brace/s when walking without assistance	[q19] ='1'
q21	Think about walking without a walking aid and without assistance. Can you walk without leg brace/s ?	• yes<br>• no, I need brace/s	[q9] ='1'
q22	Think about using 1 cane/crutch instead of no walking aids. Can you walk without leg brace/s ?	• yes<br>• no, I still need brace/s	[q21] ='0'
q23	Think about walking with 1 cane/crutch, and without leg brace/s. Can you walk without assistance?	• yes<br>• no	[q22] ='1'
q24	Think about walking with 1 cane/crutch. Can you walk without assistance (with or without leg brace/s ?	• yes<br>• no, I need assistance	[q4] ='1' or [q11] ='1' or [q11a] ='1'
q25	Think about walking with 1 cane/crutch. Can you walk with assistance of 1 person (with or without leg brace/s)?	• yes<br>• no, I need assistance of 2 people	[q24] ='0'
q26	Can you walk with 2 crutches instead of 1 cane/crutch? This includes walking with or without assistance and/or leg brace/s.	• yes<br>• no	[q25] ='0'
q27	Can you walk with a walker in any way? This includes walking with or without assistance and/or leg brace/s.	• yes<br>• no	[q26] ='0'
q28	Can you walk in parallel bars in any way? This includes walking with or without assistance and/or leg brace/s.	• yes<br>• no	[q27] ='0'
q29	Think about walking with 1 cane/crutch and assistance of 1 person. Can you walk without leg brace/s ?	• yes<br>• no, I need brace/s	[q25] ='1'
q30	Think about using 2 crutches instead of 1 cane/crutch. Can you walk without leg brace/s?	• yes<br>• no, I still need brace/s	[q29] ='0'
q31	Think about walking with 2 crutches and no leg brace/s. Can you walk without assistance?	• yes<br>• no, I need assistance	[q30] ='1'
q78	Think about walking with 2 crutches and no leg brace/s. Can you walk with the assistance of 1 person?	• yes<br>• no, I need assistance of 2 people	[q31] ='0'
q81	Think about using a walker instead of 2 crutches. Can you walk without assistance and without leg brace/s?	• yes<br>• no	[q78] ='0'

No.	Question	Answers	Branching Logic
q32	Think about walking with 2 crutches and with your leg brace/s. Can you walk without assistance?	• yes<br>• no, I still need assistance	[q78] ='1'
q76	Think about using a walker instead of 2 crutches. Can you walk without assistances and no leg brace/s?	• yes<br>• no, I need assistance	[q32] ='0'
q77	Think about using a walker. Can you walk without leg brace/s and without assistance?	• yes<br>• no, I still need assistance	[q32] ='1'
q33	Think about walking with 2 crutches and with your leg brace/s. Can you walk without assistance?	• yes<br>• no, I need assistance	[q30] ='0'
q73	Think about using a walker instead of 2 crutches. Can you walk without assistance (with or without leg brace/s)?	• yes<br>• no, I need assistance	[q33] ='0'
q74	Think about walking with a walker and without assistance. Can you walk without leg brace/s?	• yes<br>• no, I need brace/s	[q73] ='1'
q75	Think about using a walker instead of 2 crutches. Can you walk without leg brace/s and no assistance?	• yes<br>• no, I need brace/s	[q33] ='1'
q34	Think about using 2 crutches instead of 1 cane/crutch. Can you walk without assistance (with or without leg brace/s)?	• yes<br>• no, I need assistance	[q29] ='1'
q35	Think about waking with 2 crutches and without assistance. Can you walk without leg brace/s?	• yes<br>• no, I need brace/s	[q34] ='1'
q36	Think about walking with 1 cane/crutch and without assistance. Can you walk without leg brace/s?	• yes<br>• no, I need brace/s	[q24] ='1'
q37	Think about walking with 2 crutches instead of 1 cane/crutch. Can you walk without leg brace/s?	• yes<br>• no, I still need brace/s	[q36] ='0'
q38	Think about walking with 2 crutches and without leg brace/s. Can you walk without assistance?	• yes<br>• no, I need assistance	[q37] ='1'
q39	Think about walking with 2 crutches. Can you walk without assistance (with or without leg brace/s)?	• yes<br>• no, I need assistance	[q12] ='1' or [q5] ='1' or [q26] ='1' or [q12a] ='1'
q40	Think about walking with 2 crutches. Can you walk with assistance of 1 person (with or without leg brace/s)?	• yes<br>• no, I need assistance of 2 people	[q39] ='0'
q41	Can you walk with a walker instead of 2 crutches? This includes walking with or without assistance and/or leg brace/s.	• yes<br>• no	[q40] ='0'
q42	Can you walk in parallel bars in any way? This includes walking with or without assistance and/or leg brace/s.	• yes<br>• no	[q41] ='0'
q43	Think about walking with 2 crutches and with assistance of 1 person. Can you walk without leg brace/s?	• yes<br>• no, I need brace/s	[q40] ='1'
q44	Think about walking with a walker instead of 2 crutches. Can you walk without leg brace/s?	• yes<br>• no, I still need brace/s	[q43] ='0'
q45	Think about walking with a walker and without leg brace/s. Can you walk without assistance?	• yes<br>• no, I need assistance	[q44] ='1'
q80	Think about walking with a walker and without leg brace/s. Can you walk with assistance of 1 person?	• yes<br>• no	[q45] ='0'
q46	Think about using a walker instead of 2 crutches. Can you walk without assistance and no leg brace/s?	• yes<br>• no, I need assistance	[q43] ='1'

No.	Question	Answers	Branching Logic
q47	Think about walking with a walker and with your leg brace/s. Can you walk without assistance?	• yes<br>• no, I need assistance	[q44] ='0'
q48	Think about walking with 2 crutches and without assistance. Can you walk without leg brace/s?	• yes<br>• no, I need brace/s	[q39] ='1'
q49	Think about using a walker instead of 2 crutches. Can you walk without leg brace/s?	• yes<br>• no, I still need brace/s	[q48] ='0'
q50	Think about walking with a walker and without leg brace/s. Can you walk without assistance?	• yes<br>• no, I need assistance	[q49] ='1'
q51	Think about using a walker. Can you walk without assistance (with or without leg brace/s)?	• yes<br>• no, I need assistance	[q13] ='1' or [q6] ='1' or [q27] ='1' or [q41] ='1' or [q13a] ='1'
q52	Think about using a walker. Can you walk with assistance of 1 person (with or without leg brace/s)?	• yes<br>• no, I need assistance of 2 people	[q51] ='0'
q53	Can you walk in parallel bars in any way? This includes walking with or without assistance and/or leg brace/s.	• yes<br>• no	[q52] ='0'
q54	Think about using a walker and assistance of 1 person. Can you walk without leg brace/s?	• yes<br>• no, I need brace/s	[q52] ='1'
q55	Think about walking with a walker and without assistance. Can you walk without leg brace/s?	• yes<br>• no, I need brace/s	[q51] ='1'
q56	Can you walk in parallel bars without assistance (with or without leg brace/s)?	• yes<br>• no, I need assistance	[q14] ='1' or [q53] ='1' or [q7] ='1' or [q28] ='1' or [q42] ='1' or [q14a] ='1'
q57	Think about using parallel bars. Can you walk with assistance of 1 person (with or without leg braces)?	• yes<br>• no, I need assistance of 2 people	[q56] ='0'
q58	Think about using parallel bars. Can you walk the length of 10 meters (with assistance of 2 people, with or without leg brace/s)?	• yes<br>• no, I can not walk 10 meters. I can only take several steps.	[q57] ='0'
q59	Think about using parallel bars and having assistance of 1 person. Can you walk without leg brace/s?	• yes<br>• no, I need brace/s	[q57] ='1'

Table 2: The scoring matrix for SR-V2 (see Table 1 for the questions) where "no" is coded as "0", and "yes" is coded as "1".

SCORE	CRITERIA
0	[q1] ='0'
0	[q7] ='0'
0	[q8] ='0'
0	[q14] ='0'
0	[q14a] ='0'
0	[q28] ='0'
0	[q42] ='0'
0	[q53] ='0'
1	[q8] ='1'
1	[q58] ='0'
2	[q58] ='1'
3	[q59] ='0'
4	[q59] ='1'
5	[q56] ='1'
6	[q54] ='0'
7	[q80] ='0'
7	[q47] ='0'
8	[q80] ='1'
8	[q54] ='1'
9	[q47] ='1'
9	[q55] ='0'
10	[q81] ='0'
10	[q73] ='0'
10	[q74] ='0'
11	[q76] ='0'
11	[q46] ='0'
12	[q77] ='0'
12	[q75] ='0'

SCORE	CRITERIA
12	[q49] ='0'
12	[q50] ='0'
13	[q81] ='1'
13	[q76] ='1'
13	[q77] ='1'
13	[q74] ='1'
13	[q75] ='1'
13	[q45] ='1'
13	[q46] ='1'
13	[q50] ='1'
13	[q55] ='1'
14	[q34] ='0'
14	[q35] ='0'
15	[q37] ='0'
15	[q38] ='0'
16	[q31] ='1'
16	[q35] ='1'
16	[q38] ='1'
16	[q48] ='1'
17	[q20] ='0'
17	[q19] ='0'
18	[q23] ='0'
18	[q22] ='0'
19	[q20] ='1'
19	[q23] ='1'
19	[q36] ='1'
20	[q21] ='1'
