# Supplementary material for: A therapist-administered self-report version of the Walking Index for Spinal Cord Injury II (WISCI): a psychometric study
Source: Spinal Cord. 2024 Apr 2;62(6):307–13. doi: 10.1038/s41393-024-00985-8 (PMC11199132; doi:10.1038/s41393-024-00985-8)
Supplement: Supplementary file 4 [file 41393_2024_985_MOESM4_ESM.pdf]

Sub-group analysis by language:

Intraclass Correlation Coefficients ($ICC_{3,1}$):

	**Self-report WISCI completed in English. N=39**<br><br>**ICC (95% CI)**	**Self-report WISCI completed in Italian. N=41**<br><br>**ICC (95% CI)**
Reliability of SR-V1 (day 1 vs day 2)	0.78 (0.62 to 0.88)<br>0.85 (0.74 to 0.92)*	0.91 (0.85 to 0.95)
Reliability of SR-V2 (day 1 vs day 2)	0.89 (0.8 to 0.94)	0.86 (0.75 to 0.92)
Validity of SR-V1 (SR-V1 vs face-to-face WISCI)	0.85 (0.73 to 0.92)	0.94 (0.89 to 0.97)
Validity of SR-V2 (SR-V2 vs face-to-face WISCI)	0.90 (0.81 to 0.94)	0.91 (0.83 to 0.95)

*These are the results when the data of one outlier was removed. This participant scored himself as a "17" on day 1, and "2" on day 2: a difference of 15 points between the two measures. This probably reflects either an error or a misunderstanding by the participant.

Percent close agreements:

Percent close agreement between the self-report measures completed on day 1 and day 2.

Difference (points)	SR-V1 in English N=39	SR-V1 in Italian N=41	SR-V2 in English N=39	SR-V2 in Italian N=41
0	54%	63%	51%	66%
1	59%	83%	56%	78%
2	67%	85%	62%	78%
3	79%	85%	82%	85%
4	87%	88%	92%	88%
5	90%	90%	97%	90%

Percent close agreement between the self-report measurement for SR-V1 and SR-V2 (mean of scores obtained on day 1 and day 2) and face-to-face WISCI scores.

Difference (points)	SR-V1 vs face-to-face in English, N=39	SR-V1 vs face-to-face in Italian, N=41	SR-V2 vs face-to-face in English, N=39	SR-V2 vs face-to-face in Italian, N=41
0	36%	41%	41%	46%
1	46%	66%	46%	61%
2	67%	68%	74%	68%
3	77%	78%	85%	78%
4	79%	85%	85%	83%
5	87%	98%	92%	90%
